# Osteoinductive effects of preoperative dexamethasone in human dental pulp stem cells primary culture

**DOI:** 10.4155/fsoa-2016-0083

**Published:** 2017-04-03

**Authors:** Rani da Cunha Moretti, Monica Talarico Duailibi, Paulo Oliveira Martins, Jennifer Adriane dos Santos, Silvio Eduardo Duailibi

**Affiliations:** 1 CTCMol, Center of Cellular & Molecular Therapy, UNIFESP- Universidade Federal, de São Paulo- Escola Paulista de Medicina, São Paulo, Brazil; 2 Translational Surgery, Surgery Department, UNIFESP- Universidade Federal de São Paulo- Escola Paulista de Medicina, São Paulo, Brazil; 3 National Institute of Science & Technology, Biofabrication Institute, BIOFABRIS, Campinas, São Paulo, Brazil; 4 Oral & Maxillofacial Surgery Department, Hospital Ipiranga, São Paulo, Brazil

**Keywords:** dexamethasone, hDPSC, human dental pulp stem cells, third molar, tissue engineering

## Abstract

**Aim:**

The use of dexamethasone (DEX) in mesenchymal cell culture induces osteoblastic differentiation and, consequently, formation of mineralized tissues. Tissue engineering proposes the development of therapeutic strategies aimed at structural and functional regeneration of biological tissues. In this sense, cell characterization *in vitro* is critical to ensure the development of such techniques. Our objective was to evaluate the osteoinductive effect of DEX administered as a preoperative medication in primary cell culture of human dental pulp stem cell.

**Methodology:**

Cells from the third molar pulp were divided into two experimental groups, each with two preoperative medication protocols used in dental practice and differentiated by the intake of DEX in one of them. The assessment of proliferation, differentiation and viability through trypan blue, methylthiazol tetrazolium, and von Kossa and alizarin red assays, respectively, were held within fixed intervals: 7, 14, 21 and 28 days.

**Conclusion:**

This study has shown that DEX may influence *in vitro* human dental pulp stem cell behavior.

## Lay abstract

Dexamethasone (DEX) is often used as a preoperative drug in dental surgeries because it reduces pain and has favorable effects on other symptoms caused by the surgery. Additionally, when used in cell culture, its osteoinductor effect is observed. *In vitro* cell characterization is critical to ensure the development of therapeutic strategies used in tissue engineering. In this sense, this study used two preoperative medication protocols regularly used in dental practice. In Protocol A, patients did not intake DEX; in Protocol B, patients took in tablets of DEX. It was possible to assess *in vitro* behavior of human dental pulp stem cells by applying those protocols.

The development of regenerative medicine provides a viable alternative to the treatment of tissue loss caused by congenital or degenerative traumatic injuries. Tissue engineering is an interdisciplinary field that seeks to regenerate biological tissues as described in studies conducted in the field of biological sciences and engineering [1,2] in order to offer the ultimate solution to organ failure [2,3]. The use and association of isolated cells and biomaterials are the main therapeutic strategies in tissue engineering, and an accurate interaction between them is essential [1]. The regenerative capacity of third molar cells and their pluripotentiality have boosted their use in regenerative medicine [4]. Furthermore, studies have proven that it is possible to develop biological tissues from these cells, which are capable of holding all the events associated with the process [5–7]. The application of the third molar cell culture technique starts immediately after the patient's tooth is extracted; some tooth development stages are more favorable for culture [8].

When an individual undergoes a more invasive procedure, an increased amount of trauma in the surgical site and surrounding tissues occurs. Thus, the use of preoperative medications in order to reduce the surgery trauma and postoperative pain becomes necessary and should be a routine in dental practice [9]. Regarding preoperative medication, it has been observed that orally administered amoxicillin, an effective antibiotic, reduces the incidence of alveolitis, improves mouth opening limitation after surgery [10] and prevents postoperative wound infection [11]. Likewise, dexamethasone (DEX), a synthetic glucocorticoid [12,13], is used as an anti-inflammatory drug and considered a long-acting steroid, whose synthesis occurs in the adrenal cortex [14]. When DEX is administered, pain reduction occurs because the drug inhibits the release of mediators, such as lymphokines, prostaglandins (PGs), serotonin and bradykinin into the injured tissue [11]. Furthermore, glucocorticoids are effective agents in reducing postoperative consequences, such as pain, swelling and trismus [9,11]. At present, it is known that DEX effects start immediately after the traumatic effects of surgery; therefore, this drug should be administered 1 h prior to the surgical procedure [11]. This glucocorticoid acts at early stages as well as late stages of the osteogenic differentiation and accelerates osteoblast maturation; however, the mechanisms are still unclear [12]. In addition to its anti-inflammatory properties, DEX is also added to culture media due to its low cost and good results as it induces osteoblast differentiation of mesenchymal stem cells (MSCs) [12] and osteogenic development [15].

Nevertheless, it is important to know whether this drug, when orally administered, will produce the same osteoinductive effect as when it is added directly to culture media. In this context, the aim of this study was to evaluate the osteoinductive effect of DEX when administered as a preoperative medication in primary cell culture of dental pulp.

## Materials & methods

This research was approved by the Universidade Federal de São Paulo (UNIFESP) Ethical Committee under number 72518. It was designed as a primary, interventional, experimental, prospective, analytic and comparative study. In accordance with this design, six third molars were obtained from three human volunteers and collected from private clinics after extraction procedure. Informed consent was obtained for all biological tissues. Inclusion criteria for teeth selection were: intraosseous third molar tooth of healthy subjects, subjects without chronic use of any drug treatment whatsoever, subjects who lacked hypersensitivity to any of the drugs used (amoxicillin and DEX), third molars in stages 2–3 of development according to Duailibi *et al*. [8] rating and those who agreed to sign the informed consent. Exclusion criteria: teeth needing removal, dental anomaly, subjects with infectious or systemic diseases, patients with either a contaminated third molar tooth or near a contaminated area with coronary exposure to the oral environment.

Volunteers underwent two surgeries so that two samples could be obtained. Thus, two surgical procedures were performed on each patient: the upper right third molar was extracted at the first surgical session, and the lower left third molar, at the second session. This way, masticatory and swallowing functions would not be affected, and postoperative recovery would be easier. At the first surgical procedure, Protocol A (no DEX) was applied; at the second surgical session, Protocol B (with DEX). In Protocol A, the drug consisted of 4 amoxicillin 500 mg tablets (antibiotics), while Protocol B was 4 amoxicillin 500 mg tablets (antibiotics) + 14 mg DEX tablet (steroidal anti-inflammatory drug [SAID]) orally 1 h prior to surgery. The two protocols in question have been used at universities and by professional dentists in their dental practice for many years now and aim to reduce the effects of postoperative trauma, such as edema, trismus, limitation of mouth opening, among others. Thus, there was no distinction among the benefits provided to the patient.

After surgery, the tooth was placed in a falcon tube with Hank's Balanced Salt Solution (LGC Biotecnologia, Brazil) medium for preservation, then transported to a Petri dish, and finally the explant technique using the tooth pulp. Following, the teeth were washed up two- to three-times with Hank's Balanced Salt Solution, and the dental pulp was removed for explant performance. The dental pulp was fragmented with scalpel blades number 13, MED BLADE^®^ (Jiangsu Xuyi Kangning Medical, China) allocated in Petri plate (Sigma-Aldrich, São Paulo, Brazil) in 1 mm dimensions. The explant pieces were placed into 6-well culture plates, and 1 ml of culture medium was added Dulbecco's Modified Eagle's medium/Ham's F12 1:1 (Ham's F-12 nutrient mixture, LGC Biotecnologia) to each well and supplemented with 5% fetal bovine serum (FBS) and 0.5% penicillin antibiotics (LGC Biotecnologia) and streptomycin (LGC Biotecnologia). Cultivation occurred until migration and full cells confluence. The culture medium was changed every 3 days using the medium previously described. The cells were maintained in an incubator at a relative humidity of 95% oxygen and 5% carbon dioxide at a temperature of 37°C, where they were grown until the second passage, reaching confluency with a required total of 41.6 × 10^6^ cells.

## Cell proliferation

Homogenization of the cell pellet from the second passage was performed in culture medium made up using successive aspirations and ejections with a pipette (Sarstedt^®^, Inc., NC, USA) and electronic pipettor Gilson^®^ (Gilson SAS, Villiers-le-Bel, France). A total of 10 ml of the concentrated cell homogenate were withdrawn with a micropipette and a disposable tip; then, they were transferred to an Eppendorf (Sarstedt, Inc.) and mixed with the same volume of trypan blue dye at 0.4% (Sigma-Aldrich), and homogenized again. Following this step, 10 μl of the colored solution were pipetted and transferred to the chamber slide for proliferation assessment. The sample was introduced in the automatic cell counter (Countess^®^, Invitrogen, São Paulo, Brazil), which calculated the total number of both living and dead cells. This procedure was performed after cell expansion and at the end of all trypsinizations. All T75 bottles (Sarstedt, Inc.) initiated with 2 × 10^6^ cells before the experimental stages.

## Cell viability assay: methylthiazol tetrazolium

We used the methylthiazol tetrazolium (MTT) (Sigma, Brazil) assay to assess cell viability because it is a reliable and effective method based on the reduction of yellow tetrazolium salts by mitochondrial reductases of metabolically active cells. Blue crystals are formed intracellularly; they are solubilized and then analyzed by UV-VIS spectrophotometry. Thus, the lower the cell viability is, the lower the MTT reduction and spectrophotometric signal will be [16]. The trypsinized cells were previously plated in 96-well plates (Sarstedt, Inc.) in a standardized amount of 1 × 10^5^ cells per well, in triplicates. Then, 195 μl culture medium, prewarmed at 37°C and 5 μl of MTT (10 mg/ml) per well were added. The cells were incubated for 2 h away from light. Supernatant was removed by inverting the microplate. Thereafter, 200 μl of DMSO (Sigma, Brasil) were added to dissolve the crystals, and the plates were shaken in an orbital vortex. Finally, absorbance reading was carried out at a wavelength of 570 nm using a spectrophotometry reader. The procedure was similar at all fixed intervals: 7, 14, 21 and 28 days.

## Mineralization assay

### von Kossa

We used the von Kossa method in this study because it allows the identification of mineralized tissues in a sample. It is based on the binding of silver (Ag^2^) to calcium. Eosin is a counter-stain that highlights the nodule. Initially, cells were plated in four 6-well plates (Sarstedt, Inc.), identified on days 7, 14, 21 and 28, 1 × 10^6^ cells per well.

At different fixed intervals of time, the three upper wells of each well plate were washed up twice with 1 ml of distilled water, and then 1 ml of 4% paraformaldehyde solution was added to each well for 15 min. Subsequently, paraformaldehyde was discarded and the 6-well plates were washed up three-times with 1 ml of distilled water; 1 ml of 5% silver nitrate was added to each well for 20 min under UV light. The 6-well plate was washed up three- to five-times with distilled water, and thiosulfate 5% was added for 2 min. The 6-well plate was washed up three- to five-times with distilled water again and 1 ml of 0.1% eosin was added for 3 min. The 6-well plate was washed up once more with distilled water and, finally, the analysis under a microscope was performed.

### Alizarin red

Alizarin Red method was adopted in this study because it also allows the identification of mineralized tissues in a sample, based on the binding of silver (Ag^2^) to calcium. It is a dye used to confirm the results found with the von Kossa method. For the dye preparation, alizarin red (Sigma-Aldrich) was diluted in a ratio of 2 g/100 mg of distilled water. Initially, cells were placed in four 6-well plates (Sarstedt, Inc.), identified on 7, 14, 21 and 28 days, 1 × 10^6^ cells per well. At different fixed time intervals, the three lower wells of each well plate were washed up three-times with phosphate-buffered saline (LGC Biotecnologia). Following, 1 ml of 10% formaldehyde was added into each well for 30 min, and then each well was washed with 1 ml of distilled water. Alizarin red was added to the wells and the plate was maintained in an orbital shaker for 10 min and then washed up with distilled water.

The calcified nodules formed after the von Kossa and alizarin red methods were analyzed macroscopically and microscopically. The images were obtained and the nodules were quantified using AxioVert^®^ 40C (Carl Zeiss, Jena, Germany) inverted optical microscopy through Software AxioVision^®^4.1 (Carl Zeiss, Jena, Germany). Values were analyzed through GraphPad Prism5^®^ (GraphPad Software, São Paulo, Brazil) statistical program using the two-way analysis of variance (ANOVA). The software ImageJ was used to measure the nodules, and the number of nodules per well was obtained for every standard size in the study. All analyses were conducted at fixed intervals of 7, 14, 21 and 28 days.

### Statistical analysis

Results were assessed by Friedman's χ^2^ test to compare the proliferation value obtained over time (7, 14, 21 and 28 days), and the test was performed separately for each group. Cell viability analysis was performed using the Prisma5 statistical program and t-test analysis. Quantification of mineralized nodules found in the sample was performed by GraphPad Prism5^®^ statistical program using the two-way ANOVA, and the nodule's size was measured using software ImageJ. The rejection level of the null hypothesis was set at 5%.

## Results

### Cell proliferation using trypan blue method

**Figure 1.**
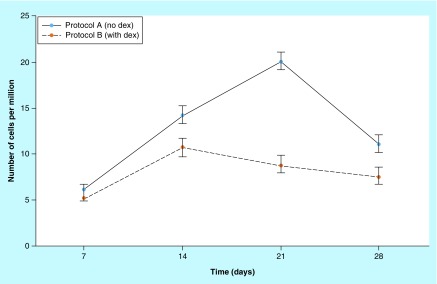
**Comparison chart between Protocols A and B.** Proliferation curves between Protocols A (no DEX) and B (with DEX), expressed as number of cells per million versus time (intervals of the analysis). In Protocol A, cell proliferation peaked on day 21 and as early as day 14 in Protocol B. DEX: Dexamethasone.

Protocol A cells (no DEX) had proliferation peak on day 21, declining thenceforth. Protocol B cells (with 4 mg DEX) had proliferation peak on day 14, declining thenceforth (Figure 1). For statistical assessment of proliferation by trypan blue, we used the Friedman's test, which is a nonparametric estimate for the comparison of the variables over time, with software BioStat. The results in Protocol A showed that cell proliferation reached a proliferation peak on day 21 of culture, obtaining a statistically significant value: p = 0.0421, while in Protocol B, proliferation was higher on day 14, also showing a statistically significant value of p = 0.0421 (Table 1).

**Table 1.** **Table showing statistical analysis of cell proliferation.**

**Days**				
**Protocol A (no dexamethasone)**				
	7	14	21	28
Sample 1	4.0	9.5	10	5.5
Sample 2	4.8	15	16	6.5
Sample 3	9.5	18	34*	21
	6.1	14.17	20.0	11.0
χ^2^ = 8.20 v		*p = 0.0421	21 dias > 7 dias	
**Protocol B (with dexamethasone)**				
	7	14	21	28
Sample 1	5.5	7.5	6.5	5.0
Sample 2	4.0	9.5	5.5	4.6
Sample 3	6.1	15*	14	13
	5.2	10.7	8.7	7.5
χ^2^ = 8.20		*p = 0.0421	14 dias > 7 dias	

The table shows the number of cells in million obtained at intervals of 7, 14, 21, and 28 days in each sample (six teeth). Protocol A (no DEX) showed cells reaching a proliferation peak on day 21 of culture and obtaining a statistically significant value: *p = 0.0421. Protocol B (with DEX) had the highest proliferation on day 14 of culture, also obtaining a statistically significant value of *p = 0.0421.

DEX: Dexamethasone.

### Human dental pulp stem cell viability assessment

**Figure 2.**
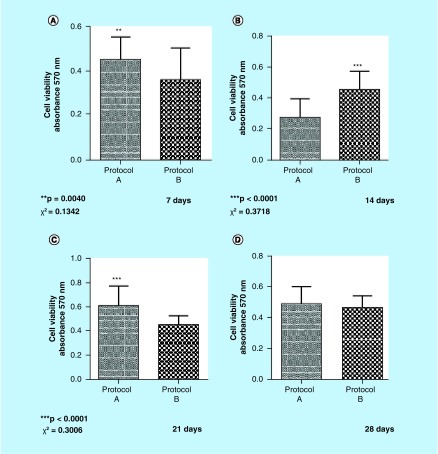
**Cell viability assay.** Viability by methylthiazol tetrazolium. The values were obtained from the analysis of *t*-test. The cells were statistically more feasible on days 7 **(A)** and 21 **(C)** in Protocol A, while Protocol B cells were statistically more viable after 14 **(B)** days of culture. **(D)** A statistically significant value was not obtained on day 28.

Following plating in 96-well plates, in triplicate, cells were read in a spectrophotometer. Analyses were performed and graphs were designed comparing groups and intervals. Statistical analysis was carried out using GraphPad Prism5^®^ statistical program. Protocol A cells (no DEX) were statistically more viable on days 7 and 21, while Protocol B cells (with DEX) were statistically more viable after day 14. On day 28, no statistically significant value was found (Figure 2).

## Mineralization assay

### von Kossa & alizarin red

**Figure 3.**
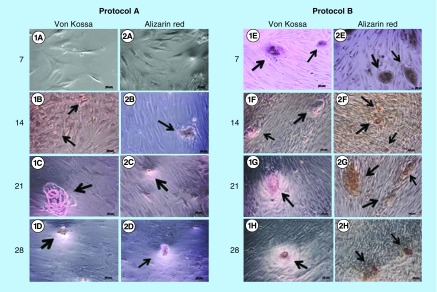
**Analysis using von Kossa and alizarin red.** Analysis between Protocols A (no DEX) and B (with DEX) regarding von Kossa and alizarin red stains, and calcified nodules formation in time slots 7, 14, 21 and 28 at 10×. On day 7, the presence of mineralized nodules was not observed in Protocol A with both von Kossa (1A) and alizarin red (2A) stains. On days 14, 21 and 28, the presence of calcified nodules (arrows) was found in Protocols A and B in both samples von Kossa (1B–1H) and alizarin red (2A–2H), bar = 50 mμ. DEX: Dexamethasone.

**Figure 4.**
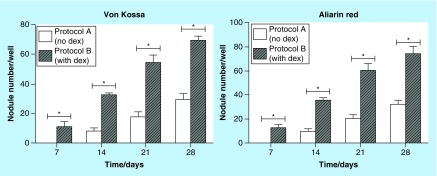
**Comparison chart of the number of nodules regarding time-nodule formation time in Protocol A (no dexamethasone) and Protocol B (with dexamethasone) with the two stains: alizarin red and von Kossa.** The numbers of nodules were counted at the fixed intervals and analyzed by GraphPad Prism5^®^ statistical program using the two-way analysis of variance. Values are presented as mean ± SD of the samples in triplicate. Statistical significance between Protocol A and B, both with von Kossa and alizarin red stains is represented as *p < 0.001. DEX: Dexamethasone.

After plating the cells in 6-well plates, von Kossa staining and alizarin red were used for examining possible mineralized nodules. In the three upper wells, we used von Kossa stain and in the three lower wells, alizarin red. This procedure was performed in all samples and were equally reproduced at intervals of 7, 14, 21 and 28 days. In Protocol A, the presence of mineralized nodule was not identified on day 7, either with von Kossa stain or alizarin red (Figure 3). On days 14, 21 and 28, we observed the formation of mineralized nodules. In Protocol B, the presence of calcified nodules was observed in all fixed intervals, namely 7, 14, 21 and 28 days, both with the von Kossa and alizarin red assays. In addition, the nodules were larger and mineralized sooner in Protocol B, on day 7, when compared with Protocol A (Table 2), which is consistent with results found in the literature [14]. The statistical analysis used for counting nodules was two-way ANOVA, and the number of nodules found in Protocol A and Protocol B was statistically significant (Figure 4), p < 000.1.

**Table 2.** **Effects of dexamethasone on nodule formation.**

**Size of nodule (μm^2^)**	**Time/days**			
	**7**	**14**	**21**	**28**
**Protocol A (no DEX)**				
von Kossa				
– 0–9999		4		
– 10,000–19,999		4	12	
– 20,000–29,999			4	3
– >30,000			4	26
**Protocol B (with DEX)**				
von Kossa				
– 0–9999				
– 10,000–19,999	7		13	7
– 20,000–29,999	3	14	10	16
– >30,000	1	9	31	47
**Protocol A (no DEX)**				
Alizarin red				
– 0–9999		3		
– 10,000–19,999	4	2	8	
– 20,000–29,999	1	12	12	
– >30,000		2	6	12
**Protocol B (with DEX)**				
Alizarin red				
– 0–9999				
– 10,000–19,999	10	7	7	10
– 20,000–29,999	2	13	16	18
– >30,000	1	16	37	46

The table shows the size mean of nodules measured by software ImageJ. The same procedure was performed both in Protocol A (no DEX) and Protocol B (with DEX) with both stains.

DEX: Dexamethasone.

## Discussion

Tooth development occurs as a result of the interactions between mesenchymal cells and epithelial ones. These interactions will first originate the enamel organ and, following, the papilla and dental follicle [17]. The other components of the tooth, such as dentin, pulp, cementum and the periodontal ligament will be developed by MSCs [17,18]. MSCs constitute a source of study in several experiments involving laboratory cell culture. Those cells have highly variable proliferation rate, depending on the formulation of the medium (basal media and supplements), the density of cell seeding, the substrate area and the physical-chemical environment [17], and may be found in various areas of the human body and in various dental tissues, among them the dental pulp of permanent teeth, also known as dental pulp stem cells, (DPSCs) [19,20]. DPSCs are capable of self-renovating and differentiating in osteoblasts, adipocytes, neural cells and odontoblasts [19,20].

In this study, we used third molar dental pulps because they are a rich source of cells used in dental science experiments. Research has shown that developmental stages 2–3 of third molar teeth have larger quantity and better quality of stem cells [8]. However, some difficulties regarding the tooth extraction technique at this stage of development often restrict the sample number and make the surgery more traumatic. In this sense, there are medications – amoxicillin and DEX – used as a protocol in dental practice that may minimize the effects produced by the surgical trauma. Amoxicillin is an effective antibiotic for reducing the incidence of alveolitis and, additionally, provides better results in mouth opening after surgery [10] and prevents postoperative wound infection. DEX is one of the most potent synthetic glucocorticoids used as an adjunctive therapy in many diseases because it has good anti-inflammatory, immunosuppressive and antiexudative properties [13,21]. It is used in several protocols, such as preoperative medication in third molar surgery due to its beneficial effects, namely inflammatory symptoms reduction, operative sequelae, such as early and late edema, trismus and pain decrease [21–23].

The use of DEX 1 h prior to surgery is justified by several studies that have evaluated its pharmacokinetics and found that its maximum effect is achieved from 1 to 2 h after oral administration [13]. Accordingly, the medication will be making the desired effect during the surgical procedure. Despite all its desirable effects, however, when systemically used in mice for a prolonged period of time, DEX produces metabolic side effects, such as insulin resistance, hypertension, glaucoma and osteoporosis [24]. Similarly, studies have shown that long-term use of DEX in humans also leads to bone loss and consequent osteoporosis [25–27] because it suppresses osteoblast function, reduces intestinal calcium absorption, increases bone resorption and suppresses endogenous gonadal steroids [26,27].

In addition to being used as a drug, DEX is widely used and added to culture media as an osteoinduction factor [12,15,19,28]. Considering that tissue engineering involves applying its techniques with the aim of developing biological substitutes that will restore, improve or maintain tissue function [1], and knowing that DEX induces osteogenic development when added to the culture medium [12,15,19,28], it is of the utmost importance to understand the *in vitro* cell behavior when the patient is given that medication, whose use is included in dental practice protocols. Therefore, the main purpose of this study was to evaluate the osteoinductive effect of DEX administered as a preoperative medication in primary cell culture of hDPSCs.

The experimental design of this study was based on fundamental preliminary experiments and aimed to characterize it in terms of reproducibility and repeatability. The methodology was based on cell culture in order to characterize the cells according to incubation time, temperature, appropriate cell culture conditions, linearity and time interval analysis. The cells obtained from third molars pulp were evaluated for proliferation, viability and formation of calcified nodules. We compared cultures from the first surgical session, when patients had not received DEX, with the cell culture from the second surgical session, when patients took in DEX. Two surgical sessions had been performed with a 15-day interval between surgeries for the same patient. In order to eliminate any bias, we compared the cells of the same donor, and the medication was the only variable. Additionally, assuming that DEX is a routinely used drug in dentistry and knowing that it produces an osteoinductive effect when added to culture medium [20–24], our goal was to evaluate whether it would have an effect on the culture of cells obtained from the stem cells from third molar dental pulp. We used time intervals of 7, 14, 21 and 28 days in our study.

DEX is an SAID that influences transcription of various genes and changes mRNA synthesis, which generate the proteins that will mediate multiple physiological effects in the bone [29,30]. In the body, DEX plays an anti-inflammatory role since it acts in various inflammation events, reducing, for example, the onset of tenascin-C, present in extracellular matrix of adult individuals’ injured tissues [31]. In addition, by blocking phospholipase A2 (that is an enzyme that promotes arachidonic acid release into cell wound), the angiogenesis, synthesis of leukotrienes and PGs decrease [31,32]. PGs, which may, in some circumstances, stimulate bone resorption, are also responsible for mediating major events related to both bone formation and repair [30,33] and play an important physiological role by enhancing bone formation as a response to a mechanical stimulus, both in animals and humans [34]. Moreover, PGs, especially PGE2, are produced by osteoblasts under the stimulation of COX2, whose expression is controlled by hormones, cytokines and growth factors responsible for bone remodeling. SAIDs inhibit transcription of several cytokine genes, such as *IL-6* [30]. *IL-6* and *-11* stimulate resorption as well as bone formation because oesteoclasts, in addition to their osteolytic function, play an important role in bone growth and development [35]. In this sense, *in vivo* bone formation is clearly a complex dynamic process, involving the actions of multiple morphogens, growth factors and hormones [36].

In *in vitro* experiments, DEX has been described in several studies as an osteoinductive factor in many different cell types [12,15,19,28,37], including mesenchymal cells, human bone marrow derived stromal cells [38], fetal rat calvarial cells [39], mouse embryo derived NIH3T3 fibroblasts [14,40] and human periodontal cells [37]. However, the mechanisms describing how DEX is modulated are still unclear [28,37]. Previous studies demonstrated that glucocorticoids, including DEX, regulate gene expression in differentiating cells and induce the affinity of the glucocorticoid receptor for its target sequence in the genome [28,41,42,28]. In this sense, the steroid hormone receptors activate specific gene transcription by binding as hormone–receptor complexes to short DNA enhancer-like elements termed hormone response elements [41,42]. According to Lian and Stein, osteoblastic differentiation *in vitro* is marked by three distinct stages of cellular activity: proliferation, extracellular matrix maturation and matrix mineralization [43]. The first stage is characterized by the high mitotic capacity of progenitor cells, represented by the expression of the cell growth associated genes, *H4 histone* and *c-fos* [44]. Due to this proliferative capacity, genes are expressed at peak levels. These genes are related to extracellular matrix formation [45]. Studies have demonstrated that MSCs, once cultured in DEX-supplemented medium, start a cascade of development, defined by the acquisition of cuboidal osteoblastic morphology, transitory induction of alkaline phosphatase production, bone matrix protein mRNAs expression and biomineralization [46]. In bone marrow stromal cells, a number of compounds can induce osteoblastic differentiation, however, the earliest-known and most readily available inducer of bone marrow stromal cell differentiation is DEX, that reliably stimulates the development of many, although not all, phenotypic characteristics of osteoblasts [38,47]. However, the effects of DEX on osteoprogenitor cell proliferation and phenotypic development also depend on both the dosage and timing of treatment [12,48]

Dental pulp cells can also be induced to express odontoblast-like markers when cultures are supplemented with DEX, but there are few available data on the specific effects of this glucocorticoid on dental pulp cells [49]. In order to examine DEX influence on dental pulp cultures, studies have focused, for example, on the analysis of alkaline phosphatase levels [14], and mRNA encoding for dentin sialophosphoprotein [49], cell proliferation [12,50], mineralization potential (alizarin red method) and flow cytometry [50].

In this study, we performed proliferation assays in order to identify possible effects of DEX on third molar DPSCs. The way DEX contacted the dental pulp cells in Protocol B (with DEX) may be inferred by the fact that the drug was producing effects in the plasma, that is, its maximum effect 1–2 h after oral administration [13], during the surgical procedure. Both tissue removal and processing prevented the cells, dislodged from the explants, from suffering interferences occurring in the body, such as, enzyme action [30] and other complex dynamic processes [36]. All cells used in the different protocols are third molar cells, characterized as cells with a high level of clonogenicity and proliferation, and the ability to generate densely calcified colonies and occasional nodules [51]. In this sense, our results sustain such assertion and show differentiation in both protocols, with Protocol B (with DEX) standing out because its cells differentiated earlier.

Assessment of cell proliferation was performed using trypan blue dye [52], and the cells were counted in an automatic counter to avoid the observer's bias. The results showed that Protocol A (no DEX) cell proliferation peaked on day 21, while Protocol B (with DEX) cell proliferation peaked on the 14th day of culture (Figure 1). Friedman's ANOVA was performed and the results were statistically significant on day 21 for Protocol A and on day 14 for Protocol B (Figure 2). After the peak of proliferation, the cells in both protocols fell into decline according to the classification found in another study [53]. These results confirm other *in vitro* studies in which DEX was shown to decrease proliferation when added to primary explants [54]. In this sense, the presence of DEX is inhibitory to cell proliferation, suggesting that DEX acts to direct osteoprogenitor cells from a state of proliferation to that of matrix maturation [12].

For cell viability, we performed the *t*-test using Prisma5 program. There was a statistically significant difference in viability of Protocol A cells (no DEX intake) on days 7 and 21, whereas Protocol B (with DEX) showed a statistically significant difference on day 14 in culture (Figure 3). In order to observe cell differentiation in mineralized tissues, we used alizarin red and von Kossa stains, as described by Puchtler *et al*. [55]. Our results have shown that cells from the teeth of those patients who had received DEX 4 mg 1 h prior to surgery differentiated earlier – a 7-day culture – compared with the cells of those patients who had not taken any medication, being consistent with another study that has also examined mineralized nodules formation in pericyte cells on the first week of exposure to DEX [14].

In this sense, we can infer that the drug was still in action in blood plasma, corroborating studies that have demonstrated that the drug reaches its maximum effect between 1 and 2 h after oral intake [13]. We have also observed that there was higher mineralization in Protocol B samples (with DEX) when compared with Protocol A samples (no DEX), thus confirming previous *in vitro* studies that showed higher mineralization in samples exposed to DEX in comparison to those that did not receive DEX [12]. It is believed that mineralization is regulated by deposition of negatively charged matrix proteins, and prolonged exposure to DEX may correspond to a transition from matrix maturation to mineralization [12,45]. Our data were consistent with those from another study which observed that glucocorticoids increase the number and size of bone nodules formed in primary bone cell cultures [38] and mineralized earlier in the presence of DEX [14], Protocol B, compared with Protocol A. In addition, it was possible to relate the DEX osteoinductive effect when directly added to culture media [12,15,19,28] to the systemic application of the drug, which produced the same osteoinductive effect.

We have shown that DEX intake produced an osteoinductive effect on hDPSC. However, it is not yet known how the drug reaches the third molar region.

More importantly, our results show that it is fundamental to understand cell behavior in these conditions. Once we know that DEX intake promotes cell differentiation within 14 days in culture, all procedures aiming to obtain undifferentiated cells should be performed with cells cultured for a time period less than 14 days.

## Conclusion

The use of DEX as a preoperative medication in third molar surgery promotes cell differentiation earlier, when observed *in vitro*. For that reason, when the protocol with preoperative DEX is applied, cells must be used prior to 14 days in culture in future clinical applications.

## Future perspective

This study is a first approach toward assessing the *in vitro* effects produced by systemic DEX. Issues, such as the type of mineralized tissues (dentine, enamel, cementum, bone) that cells expressed and how DEX was capable of reaching dental pulp cells still need to be investigated.

**Executive summary**

- Dexamethasone (DEX) is widely used and added to culture media as an osteoinductor.
- Several studies exist that add DEX directly to cell culture to promote osteoinduction in various types of cells. However, no reports can be found in the literature demonstrating its effects on the cells when administered as preoperative medication.
- Unlike what can be found in the literature, this is not a study from the lab to the clinic; conversely, it is a study from the clinic to the lab, which makes it unique.
- DEX used as preoperative medication was capable of producing osteoinductor effect in human dental pulp stem cell observed *in vitro*.
- When patients took in DEX prior to third molar surgery, we observed that *in vitro* cells differentiated earlier (7 days) than the cells of the same patients when they did not take in DEX (as seen in Figure 3).
- The number of mineralized nodules was significantly higher in Protocol B (with DEX) than in Protocol A (no DEX).
- Protocol A cells reached proliferation peak on day 21 and then began to decline while Protocol B cells peaked on day 14 and started to decline thereafter.
- This preliminary study may signal that other preoperative drugs also have effects on cell culture because there is evidence of changes in cultured cell behavior that is worth investigating with other drugs.
